# Reducing the level of postoperative thrombotic complications by the combination of low molecular weight heparin and epidural anesthesia at the patients after total hysterectomy

**DOI:** 10.1186/cc11028

**Published:** 2012-03-20

**Authors:** O Tarabrin, V Dubinina, A Turenko, S Tarasenko, S Shcherbakov, D Gavrychenko, G Mazurenko

**Affiliations:** 1 Odessa National Medical University, Odessa, Ukraine

## Introduction

Each year in the world, cancer of the reproductive system is diagnosed in more than 600,000 women. In 8 to 35% of patients with cancer of the reproductive system, pulmonary embolism was the cause of death - and in 43% the background for other fatal complications.

## Methods

The results of surgical treatment of 79 patients after hysterectomy under prolonged epidural anesthesia during the period from 2008 to 2010 entered the study. The condition of hemostasis was monitored by 12 standard biochemical tests, as well as the new instrumental method - hemoviscoelastography preoperatively, intraoperatively and every day during 10 days after surgery. Prevention of thrombotic complications in group 1 (37 patients), conducted by bemiparin 3,500 units: the first injection 12 hours before surgery, then at 6 hours after the operation in the future once a day for 10 days; group 2 (42 patients) received heparin 5,000 units: the first injection 6 hours before surgery, then 6 hours after the operation, then four times per day for 10 days.

## Results

All patients included in the study before surgery had detected hypercoagulation and inhibition of fibrinolysis: increasing of MA (maximum density of the clot, fibrin-platelet constant of the blood) to 20.7% (*P *< 0.001) and ICD (intensity of coagulation drive (the intensity of clot formation)) to 15.6%; reduction of IRCL (intensity of the retraction and clot lysis) to 13.6% (*P *< 0.05) in both groups compared to normal rates. At the first day after surgery in patients treated with bemiparin (group 1) MA and ICD decline to 12.7 (*P *< 0.05) and 9.6% (*P *< 0.001) respectively, and IRCL increased by 4.6% (*P *< 0.05) compared with preoperatively. In group 2 there was a similar picture: the reduction of MA and ICD to 10.3 (*P *< 0.001) and 6.6% (*P *< 0.05) respectively, and IRCL increased by 4.4% (*P *< 0.001). At the fifth day the condition of hemostasis in both groups came almost to the same value - a moderate hypocoagulation, normal activity of fibrinolysis. At 7 days of the postoperative period, thrombotic complications developed in one patient of the first group (2.70%). In the second group, complications developed in four (9.52%) patients: in three cases deep venous thrombosis, and in one case coagulopathic bleeding.

## Conclusion

Using a combination of bemiparin and epidural anesthesia reduces the level of postoperative thrombotic complications, such as deep venous thrombosis, and massive bleedings in the patients after total hysterectomy. Using hemoviscoelastography enables quick identification of disorders of hemostasis in patients after hysterectomy before, during and after the surgery.

